# The Effects of a Maximal Power Training Cycle on the Strength, Maximum Power, Vertical Jump Height and Acceleration of High-Level 400-Meter Hurdlers

**DOI:** 10.2478/hukin-2013-0012

**Published:** 2013-03-28

**Authors:** Carlos Balsalobre-Fernández, Carlos Mª Tejero-González, Juan del Campo-Vecino, Dionisio Alonso-Curiel

**Affiliations:** 1 Autonomous University of Madrid. Department of Physical Education, Sport and Human Movement. Madrid. Spain.

**Keywords:** resistance training, high-level hurdlers, muscular strength, athletics, testing

## Abstract

The aim of this study was to determine the effects of a power training cycle on maximum strength, maximum power, vertical jump height and acceleration in seven high-level 400-meter hurdlers subjected to a specific training program twice a week for 10 weeks. Each training session consisted of five sets of eight jump-squats with the load at which each athlete produced his maximum power. The repetition maximum in the half squat position (RM), maximum power in the jump-squat (W), a squat jump (SJ), countermovement jump (CSJ), and a 30-meter sprint from a standing position were measured before and after the training program using an accelerometer, an infra-red platform and photo-cells. The results indicated the following statistically significant improvements: a 7.9% increase in RM (Z=−2.03, p=0.021, δ_c_=0.39), a 2.3% improvement in SJ (Z=−1.69, p=0.045, δ_c_=0.29), a 1.43% decrease in the 30-meter sprint (Z=−1.70, p=0.044, δ_c_=0.12), and, where maximum power was produced, a change in the RM percentage from 56 to 62% (Z=−1.75, p=0.039, δ_c_=0.54). As such, it can be concluded that strength training with a maximum power load is an effective means of increasing strength and acceleration in high-level hurdlers.

## Introduction

Strength training has become an essential method for optimizing athletic performance, especially in sports where explosive strength and speed are key determinants (Baker and Newton, 2008; Cronin and Sleivert, 2005; Lopez-Segovia et al., 2010; Marques, 2010). In light of this, numerous authors have studied the strength changes produced in different athletic activities (e.g. jumps, sprints, or maximum repetitions) as a result of power-centered training (Cormie et al., 2010; Hunter and Marshall, 2002; May et al., 2010; McBride et al., 2002; Mujika et al., 2009; Pui-Lam et al., 2010; Rahimi and Behpur, 2005; Tricoli et al., 2005; Turbanski and Schmidtbleicher, 2010). Research on short distance running is still, however, scarce.

Satkunskiene et al. (2009) studied the changes produced in seven high-level sprinters after eight weeks of power training and found that their stride length and frequency, speed over a 40-meter sprint, and other biomechanical variables related to running technique improved significantly in these athletes. However, the training intensity of these athletes was unclear and not well individualized. Similarly, in another study with young elite sprinters, Blazevich and Jenkins (2002) highlighted the benefits of power training in terms of variables such as 20-meter sprints or maximal strength in the squat. Thus, after a seven-week training program based on lower-limb exercises (i.e. squats, knee extensions and flexions, and hip extensions and flexions) at 30–50% of one repetition maximum (1RM), the performance of these athletes improved significantly in terms of the 20-meter sprint, squat, strength, and isokinetic hip extension and flexion torque at high velocity (i.e., 4.74 rad.s^−1^).

Despite the fact that the maximum power generated by athletes occurs in highly variable 1RM percentage intervals, which may range from 30 to 80% of the 1RM depending on the training level, the training loads used in most of these studies consider the RM percentage to be a measure of *intensity* rather than an indication of the *power* produced in the training exercises (Dayne et al., 2011; Manning et al., 1986; Stone et al., 2003). As a result, measurement of the 1RM percentage at which maximum power is generated becomes essential when it comes to optimizing the intensity of power training in order to ensure that maximum power stimuli provide greater benefits in explosive sporting activities (Harris et al., 2000).

The training effects of individualized maximum power loads on short sprint performance have seldom been studied. Furthermore, although the athletic specificity of hurdling has been widely studied (Adamczyk, 2009; Iskra and Walaszczyk, 2003; Trzaskoma et al., 2010; Zang and Song, 2001; Zouhal et al., 2010), to the best of our knowledge there has been no research into the effects of power training in hurdlers. In addition, although muscle power may not be the main factor in 400-meter hurdles, it is well known that improved strength has a highly beneficial effect on performance in this type of race (Alejo, 1993; Iskra, 1991; 2012). In light of the above, the purpose of the present study was to determine the effects of maximal power training on maximum strength, maximum power, vertical jump height and acceleration in 400-meter hurdlers. The specific hypothesis was that individualized maximum strength training twice a week for a ten-week period would significantly improve all these variables in elite hurdlers.

## Material and Methods

### Participants

The sample consisted of seven male, high-level hurdlers (age: 21.7 ± 2.4 years; body mass: 75.1 ± 4.1 kg; body height: 181.8 ± 3.9 cm; personal record: 54.78 ± 2.54 s). All subjects were highly competitive athletes with national and international success and were selected at a Performance Centre in Spain. Non-random, incidental sampling by viable access was used. The study was carried out in accordance with the Declaration of Helsinki and all subjects collaborated voluntarily. All study procedures were approved by the corresponding research ethics committee.

### Design

Due to the small number of high performance athletes available and their varying competitive schedules, it was not possible to include a control group in the experimental design. As such, the study’s design was pre-experimental, with a single group and repeated measures (before and after the training cycle). The treatment was applied twice a week for ten weeks, between February and April 2011, during the European under-23 athletics championship season. The dependent variables were 1 repetition maximum (1RM) in the half squat (kg), maximum power in the jump squat (W), flight time in the squat jump (ms), flight time in the countermovement jump (ms), and 30-m sprint from a standing position (s).

#### Instruments

An Olympic bar with different plates was used to measure the maximum power of the athletes using a MyoTest Pro accelerometer (Myotest SA, Sion, Switerzland) (Crewther et al., 2011). This type of instrument is commonly used to estimate power output by taking into account both the individual’s body mass and the external load, as recommended in the scientific literature (Dugan et al., 2004). An infrared Optojump platform (Microgate Corporation, Bolzano, Italy) was used to measure explosive strength (Glatthorn et al., 2011). Speed measurements were performed using a RaceTime 2 Light kit (Microgate Corporation, Bolzano, Italy).

### Training cycle

All subjects trained twice a week (Mondays and Wednesdays) for a full cycle of ten weeks, concentrating on their lower limbs. This training consisted of five sets of eight jump squats, with a recovery period of three minutes between sets. These exercises were performed with no countermovements and with the maximum individual load at which each athlete produced maximum power. Athletes were asked to perform the jump squats as fast as possible and performed their usual training routines, none of which consisted of strength exercises, on the remaining days. All training sessions were supervised by experienced coaches.

### Measurement of the variables: sequence and protocol

A pre- and a post-test were carried out before and after the training cycle. These measurements were performed at the same time of day and within one week after a national competition so as to ensure that the athletes were in equivalent competitive shape. Coincidentally, the weather conditions and the athletes’ state of hydration and rest were similar on evaluation days. Moreover, all measurements were undertaken according to the protocol proposed by the *National Strength and Conditioning Association* (NSCA) (Earle and Baechle, 2008).

The physical tests began following a general warm-up of about 20 minutes that included light aerobic exercises, stretching and basic low-demand plyometric exercises. After weighing and measuring the athletes, the flight time of the squat jump (ms) and the flight time of the countermovement jump were measured. Each type of the jump was performed twice, with the best of the two results being recorded. Subsequently, and also according to the NSCA protocol, the 1RM of a half squat (kg) was also measured. Once the 1RM had been achieved, and after 10 minutes of recovery, the maximum power produced in the jump squat exercise (W) was measured. This exercise started at 40% of the 1RM for that day and was increased by 5% in each series until reaching maximum power. According to the protocol for the Myotest Pro (Myotest SA, Sion, Switzerland) accelerometer used, each squat jump series consisted of two repetitions, with no countermovement, starting from an initial position in which the thighs were parallel to the ground. Again, only the best jump was recorded. Finally, the athletes ran two 30-meter sprints from a standing start. These sprints were performed on a synthetic track (mondo class) and were performed in spikes. The better of the two results was recorded.

### Statistical analysis

The Wilcoxon test (*p* < 0.05) was used to compare the scores obtained by the athletes in the pre- and post-tests (Newell, 2009). The size effect was also estimated. In light of its suitability as a nonparametric measure of magnitude, Cliff’s delta parameter (δ_C_) was calculated (Cliff, 1993). In addition, both the percentage change and the values of statistical power were determined. Finally, graphic descriptive statistics were applied after transforming the direct scores into T scores. All these statistical analyses were performed using the SPSS statistics 20 program.

## Results

**Figure 1:**
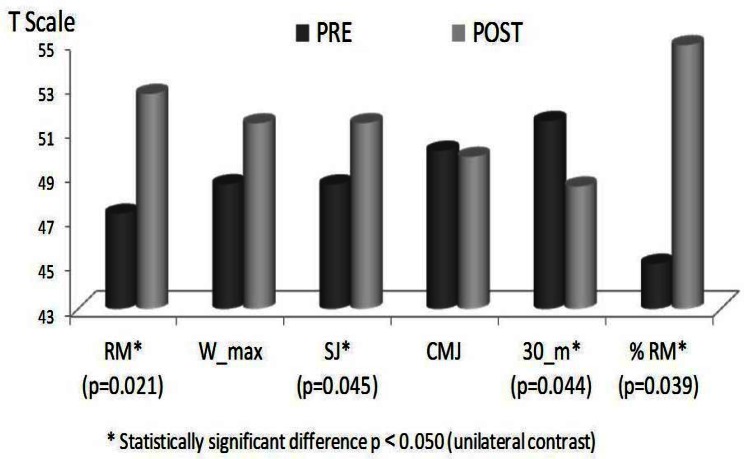
Pre/post comparison (variables transformed into T scores*). Data were transformed into T scores to homogenize the unit of measure. RM (repetition maximum), Wmax (maximum power), SJ (squat jump) CMJ, (counter movement jump), 30 m (30 meters sprint), %RM (percentage of the RM).

The results (Table 1 and Figure 1) show a statistically significant 7.9% increase in 1RM (kg) (*Z*=−2.03, *p*=0.021, Power=0.70*, δ_c_*=0.39), a 2.3% increase in flight time for the squat jump (*Z*=−1.69, *p*=0.045, Power=0.31, *δ_c_*=0.29) and a 1.43% decrease in the time required for the 30-meter sprint (*Z*=−1.70, *p*=0.044, Power=0.46, *δ_c_*=0.12). Moreover, despite not being statistically significant, a 4% increase in maximum power (W) was also observed (*Z*=−0.98, *p*=0.16, Power=0.05, *δ_c_*=0.28). In contrast, no change was observed in flight time for the countermovement jump, with values of close to −0.2% (*Z*=−0.77, *p*=0.22, Power=0.05, *δ_c_*=0.00). Finally, although not contemplated in the initial design for this study, a statistically significant 11% relative percentage change was found for the percentage of 1RM at which maximum power was generated (*Z*=−1.75, *p*=0.039, Power=0.48, *δ_c_*=0.54).

## Discussion

Our findings confirm that, after a period of maximum power training, athletes can significantly improve their maximum strength, squat and vertical jumping (greater flight time), and a 30-meter sprint performance while also improving their maximum power, although not in a statistically significant manner. Although the variables determined in the present study are not identical to those measured in previous related studies focusing on different sports disciplines (Harris et al., 2000; Lopez-Segovia et al., 2010; Manning et al., 1986; Marques, 2010; Mujika et al., 2009; Pearson et al., 2009), our results are consistent with those reported previously.

The percentages at which the athletes reached their maximum power are also worth highlighting given that this is a very important variable when it comes to establishing maximum power training loads (Bevan et al., 2010; Cronin and Sleivert, 2005; Dugan et al., 2004). Thus, the 1RM percentages in this study increased from 56 (measured before training) to 62% (measured after training), which is a statistically significant change. These values, which considerably exceed those reported for the same exercise by other authors (Bevan et al., 2010; Dayne et al., 2011; Naclerio et al., 2008; Stone et al., 2003; Thomas et al., 2007), could be due to the high training level of the subjects. Furthermore, they show that athletes can increase their maximum power and also that they are able to generate more power with each absolute and relative load, thus modifying the force-velocity relationship. This suggests an important effect on the neural mechanisms of force as the speed effort exerted in relation to high loads requires a large number of cellular motor units to be recruited, thus having a positive impact on the rate of force development (RFD) (Holtermann et al., 2007). However, since no neural adaptations have been investigated in the present study, this remains a hypothesis that could be explored in the future.

This study points to the suitability of power-based training loads (force plus speed) instead of those based simply on the displacement of maximum weight (González-Badillo and Sánchez-Medina, 2010), since, as some authors have pointed out (Kyrolainen et al., 2005), performing each repetition at maximum speed produces significant neuromuscular adaptations which will be reflected in muscular performance. As such, a relevant new research line would involve analyzing the effects of a strength training program whereby each repetition is controlled and adjusted according to predetermined optimum speed values.

Here, it is reasonable to expect that strength training would be substantially optimized since it would allow suitable maximum power loads to be specified and each repetition would be controlled to ensure it is performed at the appropriate speed. Furthermore, to achieve a greater understanding of maximum power in 400-meter hurdles, it would be interesting to determine the relationship between the power tests of this study and other tests that are metabolically more similar to those of 400-meter hurdling (e.g., 300-meter) and to analyze the changes that occur while performing the specific power training runs described in the present study.

Finally, an unexpected result of this study, namely that the countermovement jump (CMJ) of the athletes remained unchanged, should be noted. This could be due to the way in which jump squats were performed, with no advantage being taken of the stretch-shortening cycle present in the CMJ. In any case, the previous high CMJ levels for these athletes and the important improvements produced after training SJ and sprints point to the possibility that they were able to reduce their strength deficit taking advantage of their explosive force. In other words, although the CMJ remained unchanged, the SJ increased, thus showing a higher production of force per time unit with hardly any elastic contribution from the muscle. Further research will be required to confirm the concentric stimuli power effect in the stretch-shortening cycle. The high level of the subjects could also explain this result.

To summarize, despite the methodological limitations of this research (i.e., the lack of a control group and the small sample size), this study provides enough evidence to conclude that the type of power training used increases the performance of high-level hurdlers in terms of maximum strength, maximum power, vertical jump height and acceleration capability. Furthermore, as training should be individualized to achieve maximum results, this requires the specific load that produces maximum power in each athlete to be determined.

## Figures and Tables

**Table 1:** Pre- and post-test statistical measurements

Variables	*Pre M±SD*	*Post M±SD*	*Mean difference pre-post*	*% change*	*Z Wilcoxon*	*p*	*Power*	*Size (δ_c_)*
Maximum strength half squat 1 RM (kg)	172.5 ±23.9	186.2 ±26.5	13.7	7,9%	−2.03	0.021*	0.70	0.39
Maximum power JS (W)	3175 ±437	3302 ±507	127	4%	−0.98	0.16	0.18	0.28
SJ flight time (ms)	580.2 ±48.0	594.1 ±54.0	13.9	2.3%	−1.69	0.045*	0.31	0.29
CMJ flight time (ms)	602.6 ±43.9	601.3 ±53.2	−1.3	−0.2%	−0.77	0.22	0.051	0.00
30-m sprint (s)	4.19 ±0.19	4.13 ±0.16	−0.06	− 1.43%	−1.70	0.044*	0.46	0.12
% of 1RM for max power	56.25 ±4.43	62.50 ±6.54	6.25	11%	−1.75	0.039*	0.48	0.54

* Statistically significant difference, p < 0.050 (unilateral contrast)
